# Leidenfrost green synthesis method for MoO_3_ and WO_3_ nanorods preparation: characterization and methylene blue adsorption ability

**DOI:** 10.1186/s13065-023-00916-3

**Published:** 2023-02-15

**Authors:** Marwa A. Moghazy

**Affiliations:** Chemistry Department, Faculty of Science, Environmental Applications of Nanomaterials Lab., Aswan University, Aswan, 81528 Egypt

**Keywords:** Leidenfrost, Green synthesis, MoO_3_ nanorods, WO_3_ nanorods, Adsorption, Methylene blue

## Abstract

Environmental pollution is a critical issue due to its impact on humans and other organisms. An important demand nowadays is the need for a green method to synthesize nanoparticles to remove pollutants. Therefore, this study focuses for the first time on synthesizing the MoO_3_ and WO_3_ nanorods using the green and self-assembled Leidenfrost method. The XRD, SEM, BET and FTIR analyses were used to characterize the yield powder. The XRD results emphasize the formation of WO_3_ and MoO_3_ in nanoscale with crystallite sizes 46.28 and 53.05 nm and surface area 2.67 and 24.72 m^2^ g^−1^, respectively. A comparative study uses synthetic nanorods as adsorbents to adsorb methylene blue (MB) in aqueous solutions. A batch adsorption experiment was performed to investigate the effects of adsorbent doses, shaking time, solution pH and dye concentration to remove MB dye. The results demonstrate that the optimal removal was achieved at pH 2 and 10 with 99% for WO_3_ and MoO_3_, respectively. The experimental isothermal data follow Langmuir for both adsorbents with a maximum adsorption capacity of 102.37 and 151.41 mg g^−1^ for WO_3_ and MoO_3_.

## Introduction

One of the most important global issues is water pollution and the depletion of freshwater resources, which threatens economic development and ecosystem health [1, 2]. Industrial wastewater, particularly dye wastewater, is highly valued because of its high level of pollution and the challenges associated with effective treatment. A minimum of 120,000 tonnes of dyes were released into water bodies, impacting water quality, animals and plants, and the surrounding environment [3]. Methyl blue (MB) is a cationic dye, one of the most common dyes widely used as the coloring agent for cotton, paper and wood, as well as a coater for paper stock [4]. Also used in rubbers, pesticides, as a disinfector in dyestuffs, varnishes and pharmaceuticals [5]. Despite their multiple application, it causes numerous environmental issues. A tiny amount of dye in the water will impact the transparency and amount of oxygen in the water [6]. Furthermore, methylene blue is photoreactive, which means it can give reactive oxygen species when exposed to sunlight [7]. As reactive oxygen species have unpaired electrons, they are highly reactive chemically and have the potential to destroy cells in organisms. Also, because it has benzene rings, it is hard to biodegrade in nature and will accumulate in water [3, 8]. It is a toxic compound that causes many human diseases such as diarrhea, gastritis, dermatology, mutation, permanent eye damage, nausea, vomiting, mental confusion and cancer [9–11]. The removal of dye from the environment is a major requirement.

Various methods have been used for dye removal [5–7]. However, the adsorption method is still the best due to its extraordinary removal efficiency, adaptability, simplicity, recyclability and low cost [12–14]. The adsorption method for achieving high efficiency requires an adsorbent with high adsorption capacity. Many adsorbents have been used, both natural and synthetic. Of these, nanoparticles are good adsorbents due to their large area-to-volume ratio [15]. Hence, the adsorbent must exhibit high removal capabilities, fast uptake routes, and a robust mechanical structure. Metal oxide nanoparticles have extensive applications in removing different dyes because organic dyes can interact with the transition metal of the metal oxide nanoparticle [16]. Among various metal oxide nanoparticle adsorbents, MoO_3_ and WO_3_ were selected in this study.

MoO_3_ is a transition metal oxide with many applications in the field of the environmental due to its unique properties as chromogen, electronics, mechanical and catalytic properties [17, 18]. It is a common choice for water remediation applications due to its high surface area, energy storage, acid resistance, thermal stability, and low cytotoxicity [18–20].

WO_3_ is characterized by its flexibility and structural stability in a harsh environment [21, 22]. It has many environmental applications for pollutant removal due to its exceptional sensing, adsorption and/or photocatalytic properties, and is environmentally friendly [23–25].

A variety of physical and chemical methods have been used to synthesis metal oxide nanoparticles. Yet some of these methods use a lot of energy, are expensive, need expensive equipment, and are not environmentally friendly. The current trend is towards green synthetic technologies, which reduce the number of steps in the process and greatly reduce the use of costly and hazardous chemicals.

For the first time, the MoO_3_ and WO_3_ nanorods were synthesized using the Leidenfrost method. The Leidenfrost method synthesized nanoparticles through the salt solution and a heater. The technique involves forming solution droplets on a hot plate at a temperature above 200 °C (depending on the type of liquid and surface), known as the Leidenfrost temperature [26–29]. The metal oxide nanoparticle formation is illustrated as follows: a drop of water is sprinkled on a hot surface at a surface temperature above the boiling point of the water. The water drop passes through three stages as follows (1) the outer coating of the drop is evaporated as a result of touching the hot surface; (2) due to the evaporation of the outer layer of the drop, the remaining parts of the drop levitated above the hot surface which separates with a zone of the vapour; and (3) the expanded of the water drop on the hot surface leading to the fast evaporation of all droplet layers till drying leaving powder salt on the surface [28]. Understanding how metal oxide nanoparticles are formed necessitates understanding how water molecules are converted to H^+^ and OH^−^ ions via two different mechanisms. The water molecules are ionized due to (1) increasing precursor concentration, which aids in distorting water molecules' hydrogen bonds, thereby making it easier to ionize the water molecules [30]; and (2) heating the water to temperatures above its boiling point [31, 32]. In the Leidenfrost droplet, the second step (the levitated droplet), the water molecule ionized where inside the droplet, a negative charge was observed due to the predominant hydroxide ions [28]. However, the vapor has a positive charge outside the droplet due to the formation of hydronium ions. The Leidenfrost droplet is considered a reactor where the metal ion combines with the hydroxide ions forming a metal hydroxide, eventually turning into metal oxide.

This study aims to green synthesize MoO_3_ and WO_3_ by self-assembly Leidenfrost method and study the synthesis method effect on the features of the prepared nanorods, then explore the capacity of MoO_3_ and WO_3_ surfaces on the adsorption of methylene blue dye from wastewater in a comparative study.

## Experimental

### Materials

Ammonium molybdate tetrahydrate 99.5% (Koch-Light Laboratory Ltd, England), tungsten trioxide 99.8% (Alfa Aesar, Germany), methylene blue dye, NaOH (DOP, TORKIYA), NH_4_OH 28% (DOP, TORKIYE) and HCl 37% (DOP, TORKIYA) were used. All the chemicals of analytical grades were used with no further purification.

### Synthesis of MoO_3_ and WO_3_ nanorods

The Leidenfrost method was used to synthesis the adsorbent nanorods. 0.2 M molybdenum and tungsten solutions in 50 ml distilled water have been prepared. To dissolve both salts, 50 ml of NH_4_OH conc. or 50 ml of NaOH 2 M solutions were added to molybdenum or tungsten solutions. The salt solution was added drop by drop through a burette to a clean, hot beaker until a white powder of MoO_3_ and WO_3_ appeared. The resulting powder was analyzed for characterization.

### Characterization

The synthesized metal oxide nanorods were investigated by BrukerAXSD8 Germany x-ray diffraction (XRD) Radiation of Cu Kα at λ = 0.154 nm. FE-SEM (Field Emission-Scanning Electron Microscopy) was used (FE-SEM, QUANTAFEG250, The Netherlands) at 20 kV. Burnauer-Emmett-Teller (BET) Surface Area analysis was used to determine the surface area of the metal oxide nanorods beyond samples degassing at 77.35 K (Quanta CHROME NOVA 2000 Series, UK). Fourier transform-infrared (FTIR) analysis was done by Agilent Technologies, Cary 630, via a spectral transmittance measurement at room temperature. The spectral measurements were done in the range 400–4000 cm^−1^ at 2 cm^−1^ spectral resolution to characterize and investigate the variation in the functional groups of the adsorbent before and after adsorption. All the characterization was done using the powder form of the synthesized substance.

### Batch experiment

The batch experiment was done by adding different doses (0.01–0.15 g) of the metal oxide nanorods to 50 ml of various initial concentrations (10–100 ppm) of MB dye. The resultant solutions were Shacked with a speed of 200 rpm (10–60 min). The pH effect has been investigated in the range of 2–10. After equilibrium, the MB dye concentration was measured with a UV–Vis spectrophotometer (UVmini-1240 SHIMADZU) at a maximum wavelength of 660 nm.

The removal percentage and the amount of MB dye adsorbed at equilibrium q_e_ were calculated using the Eqs. (1) and (2):

1 $$\% Removal= \frac{Co-Ce}{Co}\times 100$$

2 $$qe= \frac{Co-Ce}{m}\times V$$

where C_o_ and Ce are the initial and equilibrium concentrations (mg/L), respectively. m (g) adsorbent weight and V solution volume.

## Results and discussion

### Characterization of nano adsorbents

#### XRD analysis

**Fig. 1:**
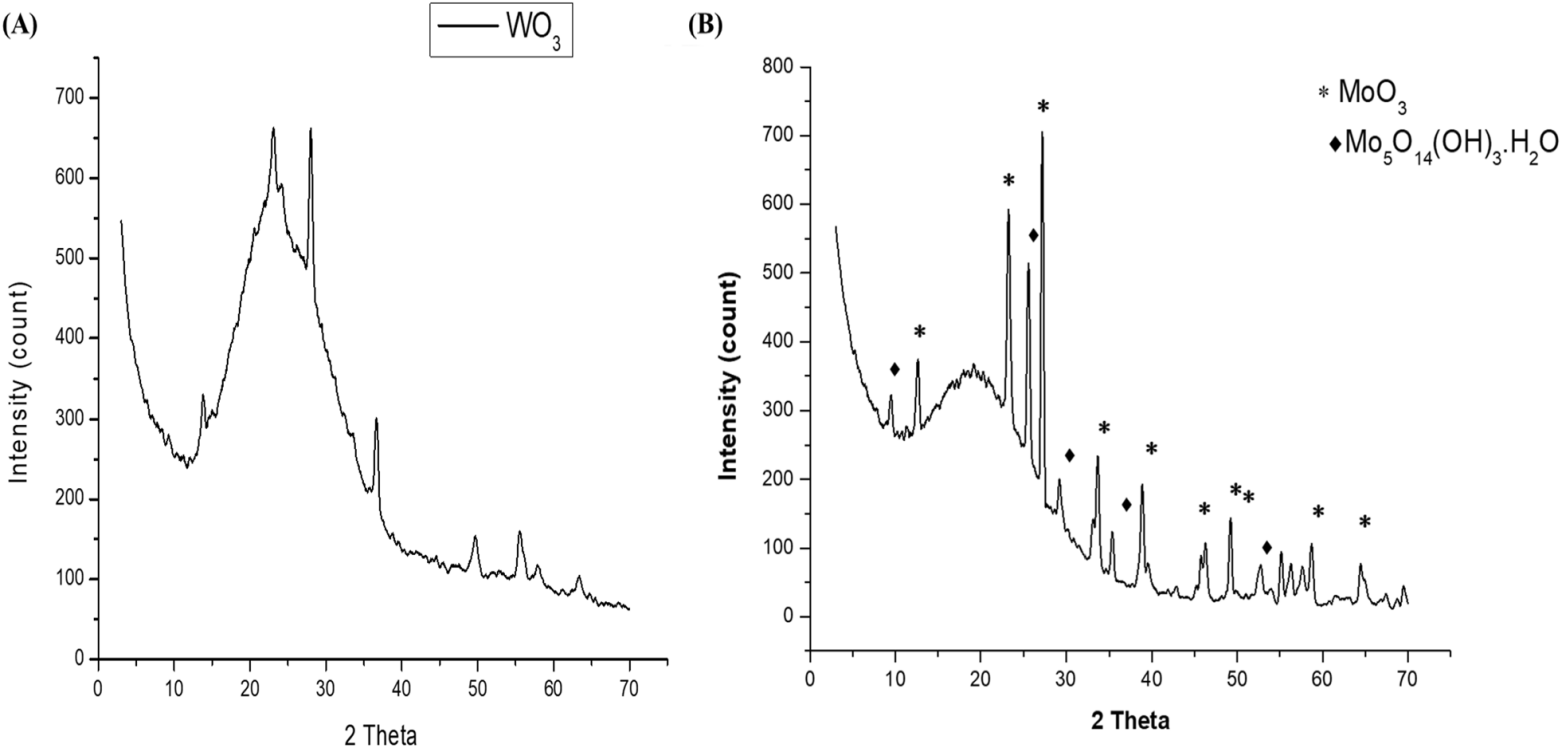
XRD pattern of (**a**) WO_3_ nanopowder and (**b**) MoO_3_ nanopowder synthesized using the Leidenfrost

A typical XRD analysis was used to characterize and indicate the compound formation of MoO_3_ and WO_3_ nanorods by the Leidenfrost method. From Fig. 1a, WO_3_ nanoparticles were formed with a hexagonal structure in a pure phase at 2 thetas, 13.82, 23.05, 28.02, 36.70, 49.71, 55.54 and 63.35^o^ with an average crystallite size 46.28 nm. Notably, no diffraction peaks other than hexagonal WO_3_ have been observed.

For MoO_3_ nanorodes, Fig. 1b, orthorhombic MoO_3_ nanoparticles were obtained at 2 theta, 12,27, 33, 45, 46, 49, 52, 54, 55, 56, 57, 58, 64, 67 and 68°. The crystallite size was determined to be 53.05 nm. A diffraction peak of the hexagonal molybdenum oxide hydroxide hydrate second phase was observed, which indicates MoO_3_ is not in a pure form. Crystallite size was calculated using the Scherer equation (Eq. 3) as follows:

3 $$D=\frac{0.9\lambda }{\beta cos\theta },$$

where D, λ, β and ϴ are crystallite size, X-ray source wavelength, the full width at half maximum of the peak and the angle at which diffraction intensity is maximized.

#### SEM analysis

**Fig. 2:**
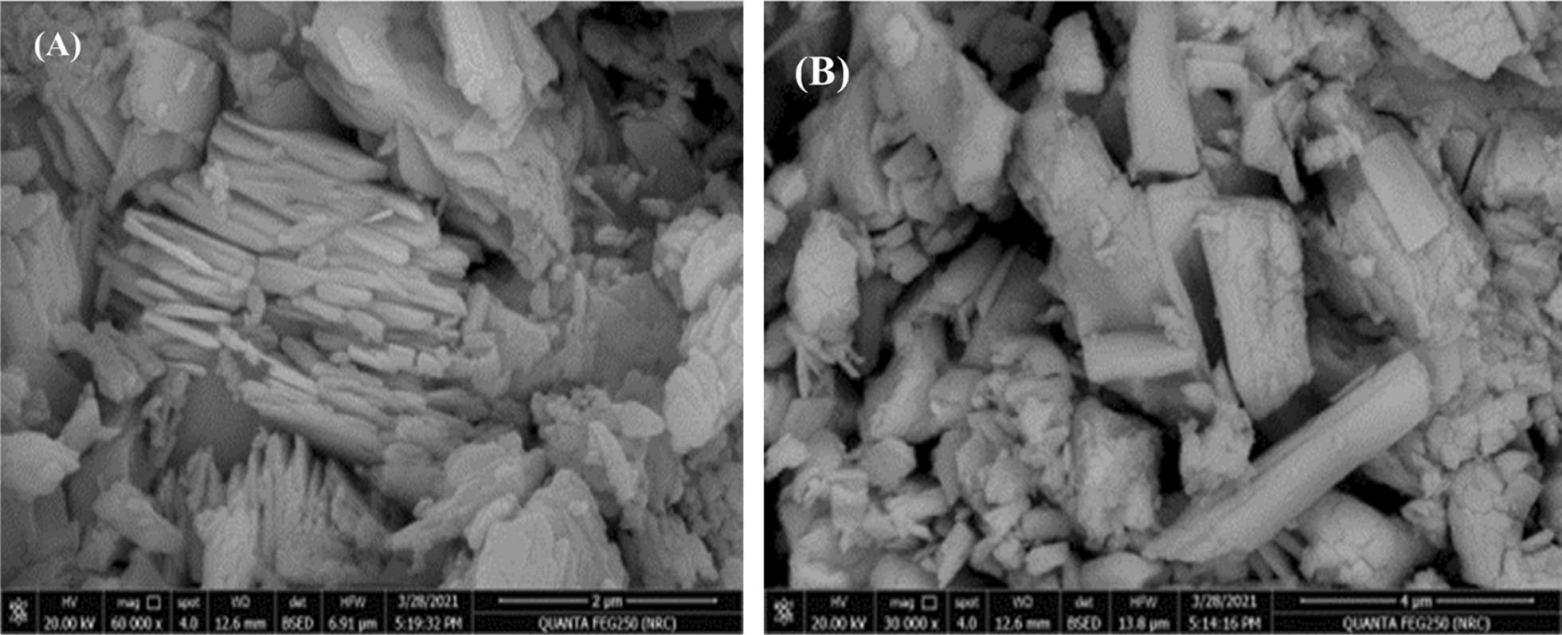
SEM morphology of (**a**) MoO_3_ nanopowder and (**b**) WO_3_ nanopowder synthesized using the Leidenfrost method

The surface morphologies of MoO_3_ and WO_3_ nanopowder were described in Fig 2a, b. For MoO_3_, a nanorods and plate-like morphology were obtained with different particle sizes. At the same time, WO_3_ exhibit a rods structure.

## Batch experimental

### Effect of adsorbent dose

**Fig. 3:**
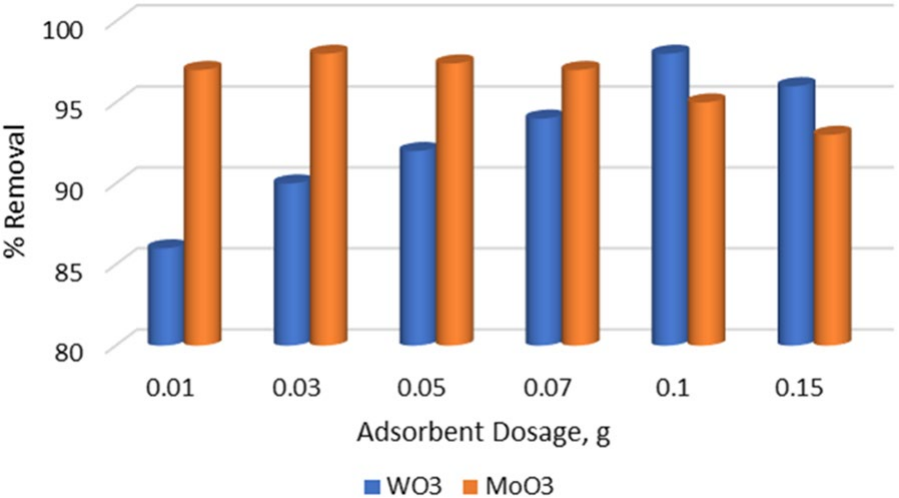
Effect of adsorbents doses for removal of 50 ppm MB using WO_3_ and MoO_3_

For both adsorbents (MoO_3_ and WO_3_), the effect of the adsorbent dose was investigated in the range of 0.01–0.15 g. Figure 3 shows that the highest elimination percentage (98%) was achieved by 0.03 and 0.1 g for MoO_3_ and WO_3_, respectively.

The percent removal of the MB dye using the WO_3_ adsorbent increased as the adsorbent dose increased (0.01–0.1 g) (86–98%). This is explained by the fact that WO_3_ has a limited surface area, 2.67 m^2^g^−1^, according to BET measurements, implying that increasing the WO_3_ dose resulted in more active sites on the WO_3_ surface. Due to the WO_3_ surface saturation, the increase in the WO_3_ amount caused the removal percentage to drop to 96%. In contrast to WO_3_, MoO_3_ surface saturation occurs faster, with maximum removal of 0.03 g and a gradual percentage reduction to a minimum of 0.15 g with 93%. In comparison, the MoO_3_ nanopowder had a higher removal % over the dose range investigated as well as a faster surface saturation with a lower dose than the WO_3_ nanopowder. This is owing to the high surface area of the MoO_3,_ which measures 24.72 m^2^g^−1^ according to BET measurements.

### Effect of shaking time

**Fig. 4:**
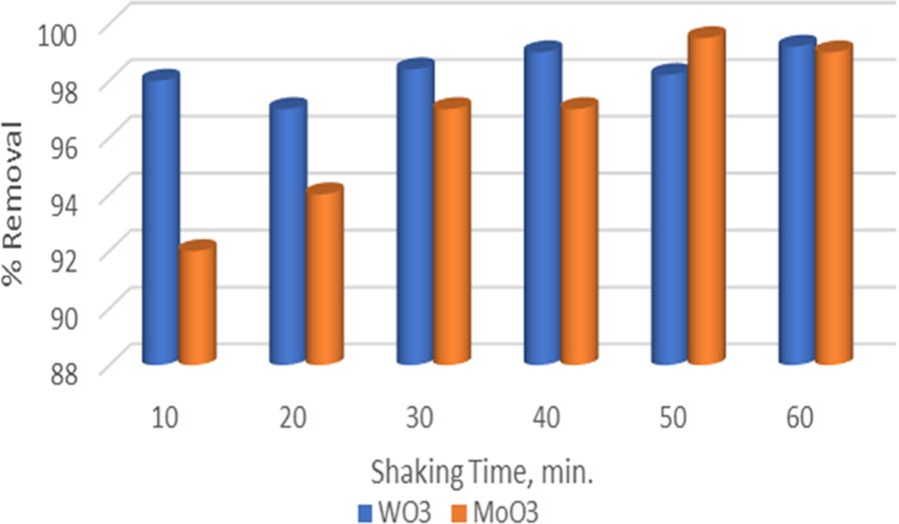
Effect of shaking time on adsorption of 50 ppm methylene blue dye using MoO_3_ and WO_3_ nanoparticles

The effect of contact time for MB dye adsorption on MoO_3_ and WO_3_ was studied in the range of 10–60 min. From Fig. 4, it is clear that the amount of the dye adsorbed using the two adsorbents is relatively fast. The WO_3_ adsorbent gives a removal percentage ranging from 98% for 10, 30, and 50 min to 99% for 40 and 60 min., so 40 min was chosen as the optimum contact time. In the case of MoO_3_ adsorbent, as illustrated in Fig. 4, a gradual increase in the contact time led to a gradual increase of the dye removal percentage to be maximum at 50 min with 99.5%.

By comparing both adsorbents, the WO_3_ surface response time of the MB dye is faster than the MoO_3_ nanorods. MoO_3_ nanopowder has a lower efficiency removal percentage along the examined contact time range than WO_3_.

### Effect of pH

**Fig. 5:**
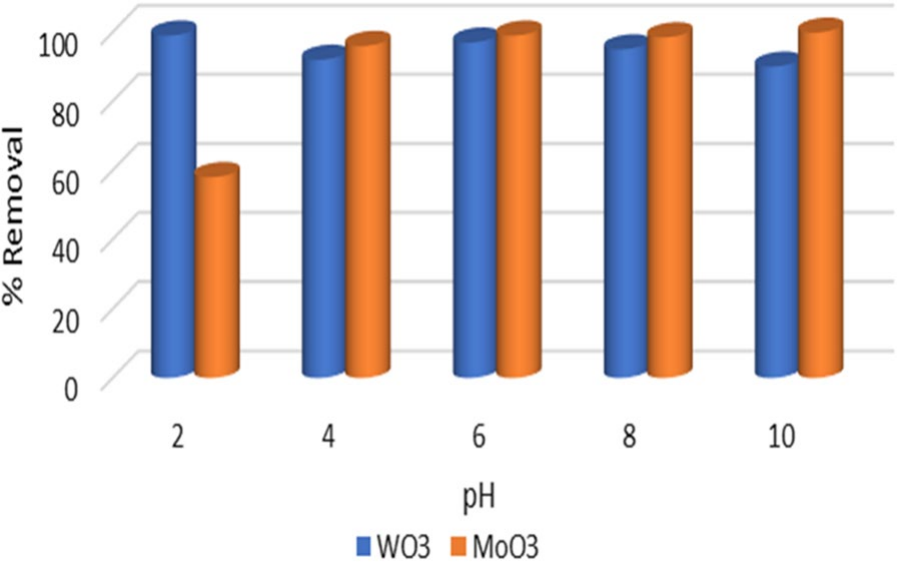
Effect of pH on adsorption process of MB using WO_3_ and MoO_3_ nanoparticles

The effect of pH is an essential parameter that needs to be investigated because of its impact on the charge of the adsorbent surface and the mechanism of adsorbent removal. pH was studied between 2 and 10 for both adsorbents in this study. From Fig. 5, for WO_3_ adsorbent, pH 2 shows 99.00%, while for MoO_3_ adsorbent, pH 10 gives 99.85% removal for the MB. These results show that the two adsorbents work in two different media where WO_3_ gives maximum removal at a very acidic medium, whereas MoO_3_ yields the highest percentage in a very basic medium.

For WO_3_ nanorods, during all studied pH, high efficiency removal of MB (≥ 90%) was observed. A high percentage of elimination was recorded at pH 2 (99%), decreasing progressively with increasing pH. This is explained by the fact that, according to the previous literature, the point of zero charge (PZC) of WO_3_ nanorods almost ranged 2.5 [24, 33–35]. Below the PZC, the WO_3_ surface has a positive charge; above PZC, the surface has a negative charge. On the other hand, the stability of the WO_3_ nanorods is affected by the pH of the solution, which dissociates in highly acidic and basic environments by the H^+^ and OH^−^ ions. Thus, in a highly acidic medium, the WO_3_ surface protonated to be as follows [33, 36] (Eqs. 4, 5)

4 $${\text{WO}}_{{{3}\left( {\text{s}} \right){ }}} {\text{ + H}}^{ + } {\text{ = WO}}_{{2}} {\text{OH}}^{ + }_{{\text{(aq)}}} .$$

5 $${\text{WO}}_{{2}} {\text{OH}}^{ + }_{{\text{(aq)}}} {\text{ + H}}^{ + } {\text{ = WO}}_{{2}}^{{2 + }} \left( {{\text{aq}}} \right){\text{ + H}}_{{2}} {\text{O}}{.}$$

Increasing the pH led to reducing the removal percentage to be minimized at pH10 by 90%. WO_2_^2+^ formed on the surface of the WO_3_ adsorbent (Eq. 5) gives the surface a positive charge and as known from the previous literature, MB dye is classified as a cationic dye that has a positive charge [8], so a repulsion force between the adsorbent surface and adsorbate is established. Above the point of zero charge, the surface is negatively charged, which facilitates the adsorption of the cationic dye through electrostatic attraction. This is attributed to that WO_3_ particles are not chemically stable in stronger alkaline solutions as they tend to dissolve due to alkaline corrosion (Eqs. 6, 7) [24].

6 $${\text{WO}}_{{{3}\left( {\text{s}} \right){ }}} {\text{ + 2NaOH = Na}}_{{2}} {\text{WO}}_{{4}} {\text{ + H}}_{{2}} {\text{O}}_{{\text{(aq)}}} {.}$$

7 $${\text{Na}}_{{2}} {\text{WO}}_{{4}} {\text{ + H}}_{{2}} {\text{O = 2 Na}}^{ + } {\text{ + WO}}_{4}^{2 - } .$$

On the other hand, for MoO_3_ adsorbent, the removal efficiency be minimum at pH 2 (58%) which increased gradually to be maximum at pH 10 (99.85%). The maximum removal was observed in very basic environments. In aqueous solutions, the metal oxide surface adsorbs water molecules that separate to OH^−^ forming M-OH. In alkaline media, deprotonation of the hydroxyl groups on adsorbent surfaces, as shown in Eq. (8) occurs due to the amphoteric performance of most heavy metal hydroxides [33]. The adsorption mechanism on the MoO_3_ surface is controlled by electrostatic attraction between the negative surface charge and the MB positive charge [37]. As a result, as the pH increases, the uptake of the dye increases to be maximum at pH 10. These results agree with Rakass, et al., which found pH 11 yields 99% removal of MB [38] and (2), et al., Jiang et al. [39] Li et al. [40] found pH 9 is an optimum condition.

8 $${\text{M}}-{\text{OH=M}} - {\text{O}} - {\text{ + H}}^{ + } {.}$$

Comparing the two adsorbents’ surfaces, WO_3_ surface has high efficiency removal through all studied pH than MoO_3_.

### Effect of dye concentration

**Fig. 6:**
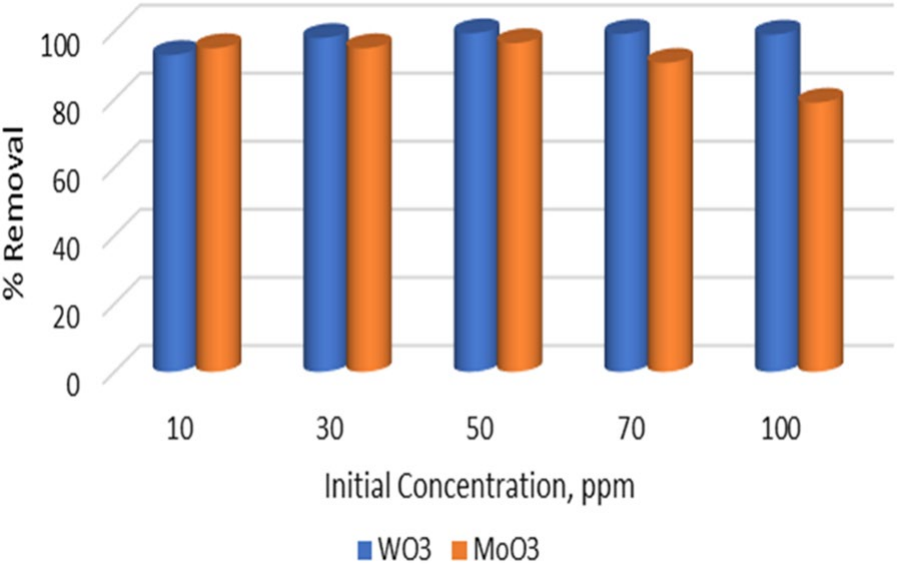
Effect of concentration on Adsorption capacity of MB Dye on WO_3_ and MoO_3_ nanoparticles

The effect of MB dye concentration on adsorption was illustrated in Fig. 6. It is clear that, or WO_3_, as the MB concentration increase, the percent removal increase to be maximum at 50 ppm with 99.4%, which become nearly constant (99%) for the higher concentrations, means that the WO_3_ surface has high vacant active sites and the capacity to adsorb to 100 ppm [41].

In the case of MoO_3_ nanopowder, Fig. 6, it was clear that 10 and 30 ppm achieve the same removal percentage (95%), which becomes a maximum at 50 ppm. Beyond 50 ppm, the percent removal decreases to be minimized (79%) at 100 ppm. Comparing MoO_3_ and WO_3_, the MB uptakes achieve a higher percentage using WO_3_ nanopowder than MoO_3_.

### FTIR

**Fig. 7:**
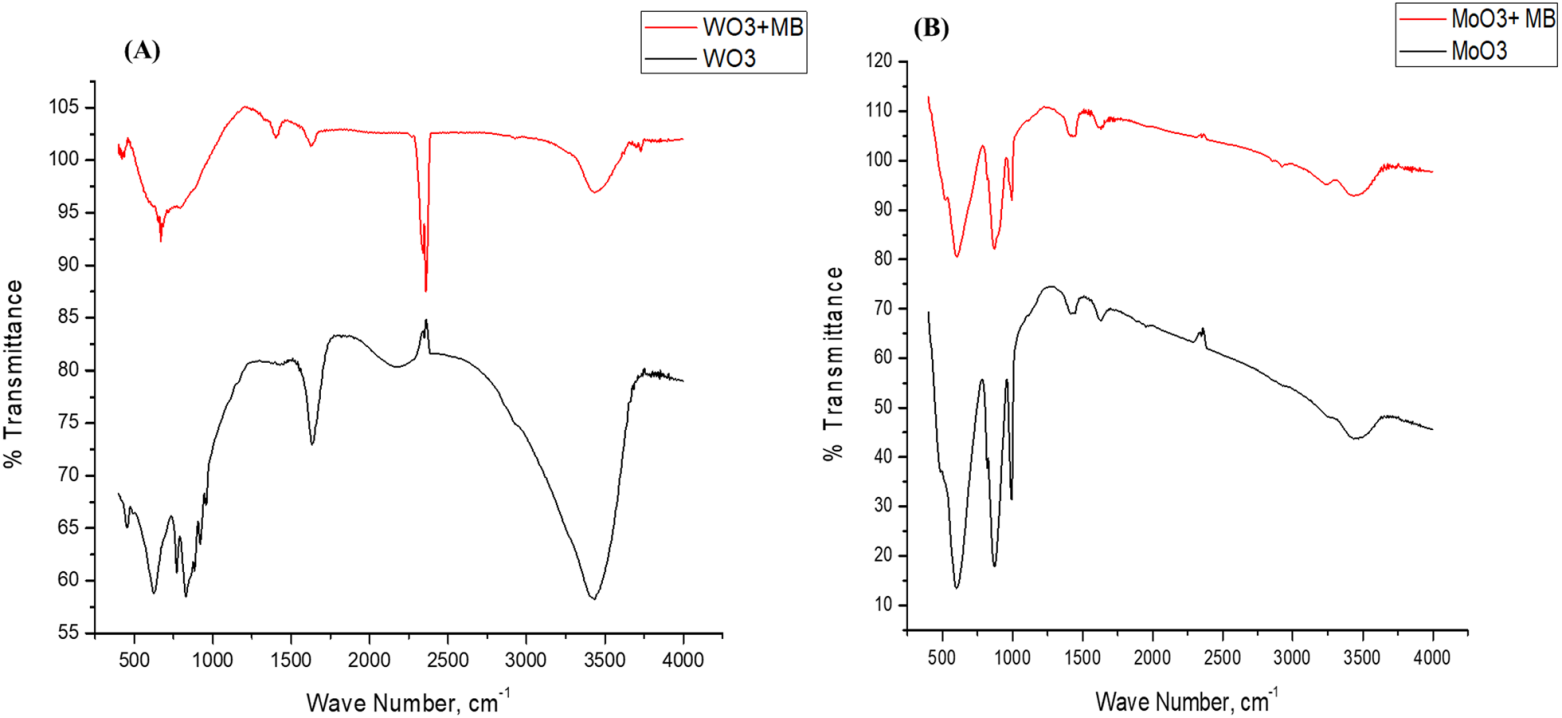
FTIR of (**a**) WO_3_ Nanoparticle before and after MB adsorption. **b** MoO_3_ nanoparticle before and after MB adsorption

The FTIR spectra of the prepared WO_3_ and MoO_3_ nanopowder exhibit a typical vibration in the range of 400–4000 cm^−1^. The WO_3_ and WO_3_ + MB FTIR are shown in Fig. 7a. Distinct bands at 624, 770 and 829 cm^−1^ were attributed to the stretching and bending vibrations for O–W–O and W–O–W in WO_3_. A vibration bands were obtained at 1639 and 3435 cm^−1^, related to OH from the H_2_O molecule [42]. On the other hand, after MB adsorption, two new peaks were formed at wavenumbers 1404 and 2360 cm^−1^, corresponding to the aromatic ring structure of MB and the C–O as well as C=O groups, respectively [43]. FTIR of WO_3_ shows bands at wavenumbers 668, cm^−1^ attributed to O–W–O of WO_3_ [44]. The vibration bands at 1625, 3434 and 3729 cm^−1^ are related to the OH group of the water molecule [45].

The FTIR of MoO_3_ and MoO_3_ + MB were represented in Fig. 7b. Four characterized peaks were observed at 600, 872, 993, and 3446 cm^−1^ for MoO_3_ nanorods. The peaks at 3446 cm^−1^ seem to arise from the O–H modes of the water of representation. The strong vibration band is observed at 600 cm^−1^, corresponding to the stretching vibrations of Mo–O–Mo [46]. The strong peaks at 872 cm^−1^ and 993 cm^−1^ indicate the stretching vibrations of Mo=O [47]. A small peak formed at 1631 cm^−1^ is related to the -OH bending vibration band. After dye adsorption, a small shift was observed for all peaks to a higher wavenumber with an enhanced peak intensity. In the case of MoO_3_ + MB, the same four basic peaks as in the MoO_3_ nanorods were formed as well as two new peaks at 1433 and 2924 cm^−1^ were observed, which related to the aromatic range in the MB dye and –CH– aromatic stretching vibration band [48].

## Isotherm study

Many different models were used for defining equilibrium experiments for the adsorption of various pollutants on solid surfaces. Freundlich and Langmuir's most popular isotherm models were applied in this study. The isotherm models illustrate the relation between the adsorbate and adsorbent and surface homogeneity.

**Fig. 8:**
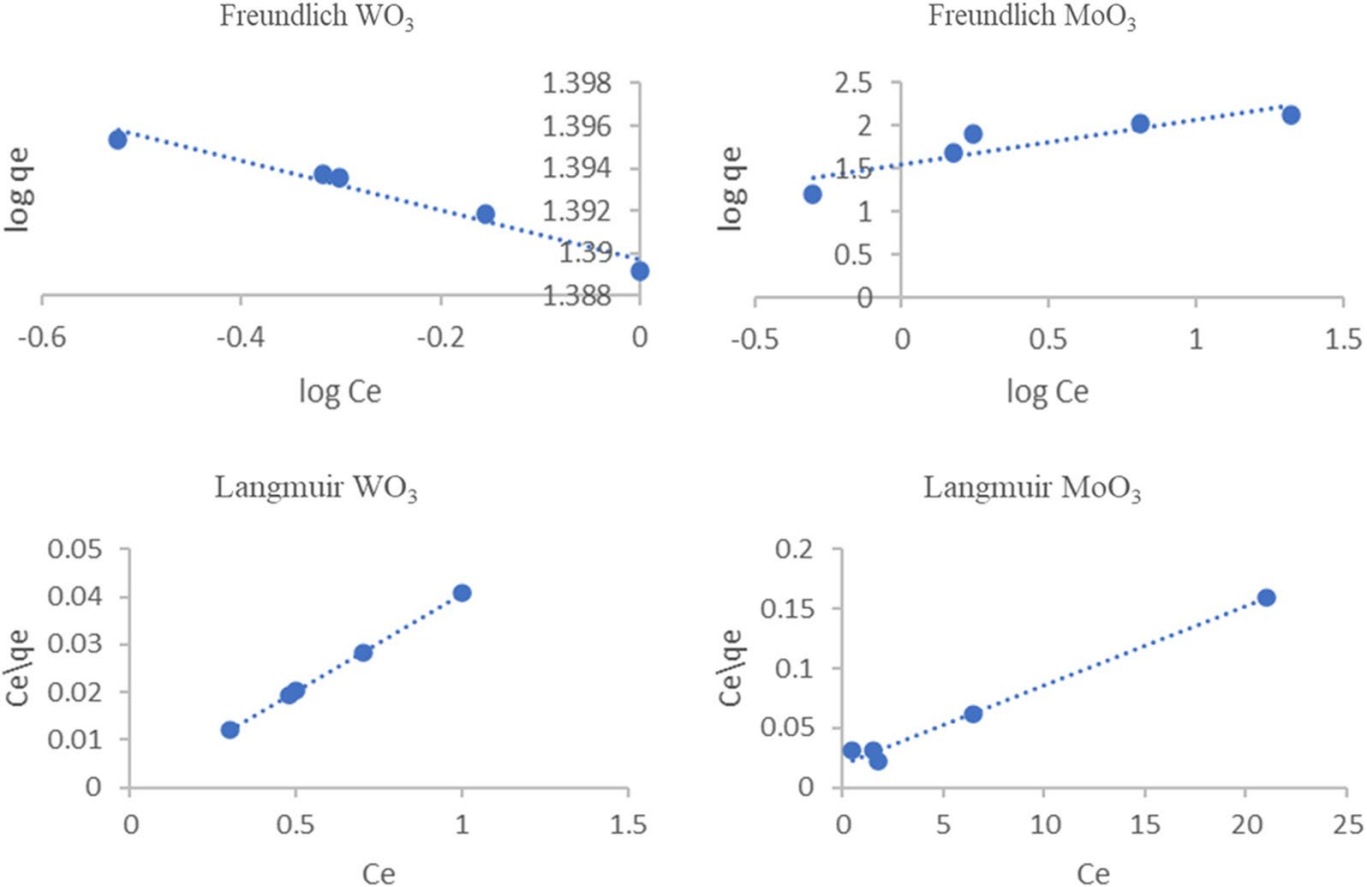
Adsorption isotherms of MB dye onto WO_3_ and MoO_3_ nanoparticles

Freundlich and Langmuir mathematical equations are described in Eqs. (9, 10, respectively), where q_e_ (mg g^−1^) the amount of adsorbate adsorbed/gram of adsorbent at equilibrium, C_e_ (mg L^−1^) the equilibrium concentration in solution, k_F_ and K_L_ are Freundlich and Langmuir constants cooperated to adsorption capacity (L/mg). 1/n an empirical value correlated to adsorption intensity. When plotting log q_e_ against log C_e_, the 1\n and log k_f_ values can be obtained from the slope and straight-line intercept [49]. For this study, the K_f_ and 1\n were shown in Table 1. Considering the R^2^ values of the two adsorbents from the isotherm models (Fig. 8; Table 1), it was obvious that the two studied adsorbents follow the Langmuir model (R^2^ = 0.99). The isotherm follows Langmuir meaning that the MB dye adsorption occurs in a homogeneous chemosorption monolayer in adsorbents surfaces. The maximum adsorption capacity (q_m_) was 102.37 and 151.41 mg g^−1^ for WO_3_ and MoO_3_ nanopowder, respectively.

**Table 1:** Isotherm model parameter of adsorption of MB dye

Adsorbent	Langmuir isotherm				Freundlich isotherm		
	q_max_	K_L_	R^2^	R_L_	1\n	K_f_	R^2^
WO_3_	102.73	0.278	0.997	0.044–0.264	− 0.012	24.53	0.962
MoO_3_	151.41	0.332	0.987	0.029–0.231	0.523	35.44	0.799

9 $$\mathrm{log}{q}_{e}= \mathrm{log}{K}_{f}+ \frac{1}{n}\mathrm{log}{C}_{e}$$

10 $$\frac{{C}_{e}}{{Q}_{e}}=\frac{1}{{q}_{m}}{C}_{e}+ \frac{1}{{q}_{m}{K}_{L}}$$

The fundamental properties of the Langmuir isotherm can be expressed in terms of a dimensionless constant known as the separation factor or equilibrium parameter, which is given by the following Eq. (11) [50].

11 $${{{R}}}_{{{L}}}=\frac{1}{1+{{{K}}}_{{{L}}}{{{C}}}_{{{o}}}}$$

where C_0_ (mg/L) is the initial MB concentration and K_L_ (L/mg) is the Langmuir constant. The value of the R_L_ illustrates the adsorption isotherm shape and favorability of the adsorption process based on the Langmuir isotherm. The nature of the adsorption process according to the R_L_ value describe as follow: if R_L_ > 1 undesirable, R_L_ = 1 linear, R_L_ = 0 Irreversible and 0 < R_L_ < 1 desirable. In this study, for the WO_3_ and MoO_3_ adsorbents the R_L_ value range in 0 < R_L_ < 1 as illustrated in Table 1 meaning that the adsorption o MB dye on the two adsorbent surfaces is desirable.

## Kinetic study of dye adsorption

**Fig. 9:**
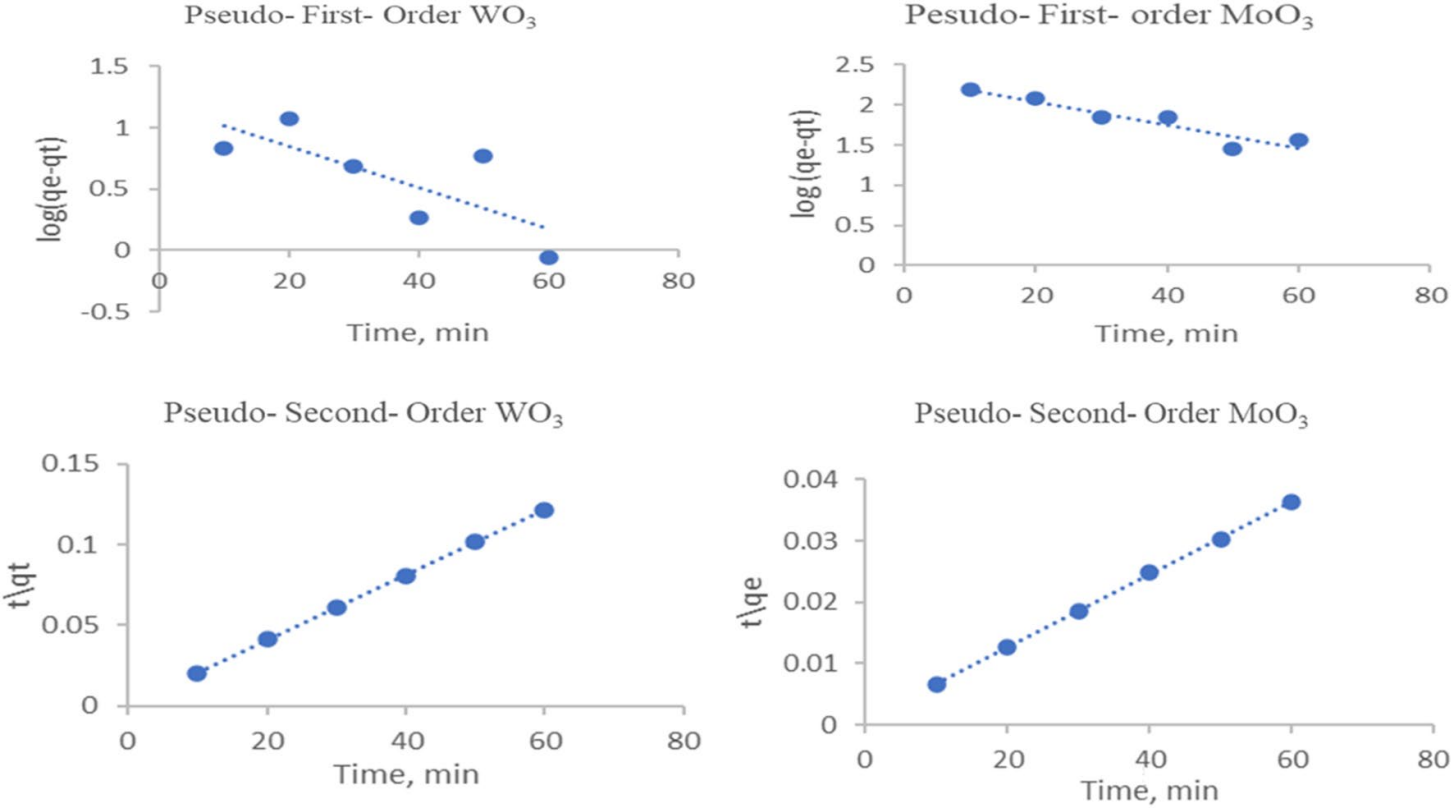
Kinetics adsorption study of MB dye onto WO_3_ and MoO_3_ nanoparticles

The kinetics of MB adsorption using WO_3_ and MoO_3_ nanorods were studied by pseudo-first-order Eq. (12) [51] and pseudo-second-order models Eq. (13) [52, 53]:

12 $$\mathrm{log}\left({q}_{e}-{q}_{t}\right)=\mathrm{log}{q}_{e}-\left(\frac{{K}_{1}}{2.303}\right)t$$

13 $$\frac{{{t}}}{{{{q}}}_{{{t}}}}= \frac{1}{{{{K}}}_{2}{{{q}}}_{{{e}}}^{2}}+\frac{{{t}}}{{{{q}}}_{{{e}}}}$$

where q_e_ (mg/g) and q_t_ (mg/g) are the capacity of the adsorbed MO on the adsorbent at equilibrium and at time t; k_1_ (min^−1^) and k_2_ (g/mg·min) are the pseudo-first-order and pseudo-second-order rate constant, respectively. T (min) is the adsorption time. In comparing the two adsorbents, Fig. 9 and Table 2, it was clear that the two adsorbents achieve the pseudo-second-order model with R^2^ values of 0.99, meaning that chemosorption adsorption occurs.

**Table 2:** Adsorption kinetic parameters of MB adsorption

Adsorbent	Pseudo-first-order		Pseudo-second-order	
	K_1_	R^2^	K_2_	R^2^
WO_3_	0.0384	0.562	0.0068	0.999
MoO_3_	0.0329	0.883	0.0005	0.999

Table 3 summarizes the findings of this study and compares them to other literature. Table 3 shows that MoO_3_ nanorods have the highest adsorption maximum capacity (q_m_) followed by WO_3_ compared to other adsorbents. Also, high adsorption removal (99%) was obtained with the two adsorbents of this study in a short time (40 for WO_3_ and 50 min for MoO_3_) relative to other studies except for activated carbon (AC) which recorded 5 min shaking time optimum equilibrium condition with 95% removal.

**Table 3:** Comparison of the adsorption best conditions of this study with other literature

Adsorbent	Dose, g\L	Shaking Time, min	pH	[Dye], ppm	%	q_m_	Reference
MoO_3_	0.6	50	10	50	99	151.41	This work
WO_3_	2	40	2	50–100	99	102.73	This work
Alg/Clin/Fe_3_O_4_	2	60	10	10	93.62	12.48	[54]
Clin/Fe_3_O_4_	1	60	10	10	97.57	45.66	[54]
Activated carbon (AC)	0.05	5	6.5–7	100	95	148.80	[55]
WO_3_	0.002	–	5	–	–	64.20	[21]
*Hordeum vulgare* bran (BB)	2.5	4 h	5.07	10	–	63.20	[56]
Enset (*Ensete ventricosum* midrib leaf, EVML)	2.5	1 h	5.07	10	–	35.50	[56]
Gum ghatti–graft–poly(4-acryloyl morpholine) hydrogel	–	3.33 h	7	1000	–	90.60	[57]
Xylan–gelatin-crosslinked hydrogel	–	5.83 h	5.84	40	–	26.04	[58]
Schott or taro tuber hydrogel	0.12	80	8.5	20	72.35	12.50	[59]
Magnetite hierarchical hollow silica spheres (Fe3O4@HHSS)	1	120	7	10	97.6	71.45	[60]
Cellulose capped magnetite nanofluids	–	–	–	–		13.54	[61]
Magnetite nanorods coated with green tea polyphenols	1	16	7	3.5	95	7.25	[62]
Iron impregnated nanoclay	0.08	120	7	40	–	79.68	[51]
Nanoclay	0.12	140	11	20	–	54.85	
nZVI	0.14	140	13	20	–	15.25	

## Conclusion

The environmentally benign Leidenfrost process synthesizes WO_3_ and MoO_3_ nanorods in succession, with crystallite diameters of 46.28 nm and 53.05 nm, respectively. Comparing the two adsorbents shows that both adsorbents have high removal capacity for MB dye removal. The adsorption equilibrium for both adsorbents follows the Langmuir model with a maximum adsorption capacity of 24.34 and 151.41 mg/g for WO_3_ and MoO_3_, respectively. The results of the kinetic study indicated that both adsorbents undergo the pseudo-second-order pattern. The adsorption equilibrium takes place in 40 and 50 min with a small dose quantity (0.1 and 0.03 g/50 ml) for WO_3_ and MoO_3_.

## Data Availability

All data included in this study are present in this published article.

## References

[1] Basheer AA (2018). New generation nano-adsorbents for the removal of emerging contaminants in water. J Mol Liq.

[2] Mohammadi A, Mirzaei A, Javanshir S (2022). Sonochemical synthesis of inorganic cryogel Ag_2_Mo_3_O_10_@Ag/AgO: structural characterization, antibacterial activity, and dye adsorption properties. RSC Adv.

[3] Andreas A, Winata ZG, Santoso SP, Angkawijaya AE, Yuliana M, Soetaredjo FE (2021). Biocomposite hydrogel beads from glutaraldehyde-crosslinked phytochemicals in alginate for effective removal of methylene blue. J Mol Liq.

[4] Liu T, Li Y, Du Q, Sun J, Jiao Y, Yang G (2012). Adsorption of methylene blue from aqueous solution by graphene. Colloids Surf B Biointerfaces.

[5] Zhang F, Lan J, Yang Y, Wei T, Tan R, Song W (2013). Adsorption behavior and mechanism of methyl blue on zinc oxide nanoparticles. J Nanoparticle Res.

[6] Pereira AGB, Rodrigues FHA, Paulino AT, Martins AF, Fajardo AR (2021). Recent advances on composite hydrogels designed for the remediation of dye-contaminated water and wastewater: a review. J Clean Prod.

[7] Khajeh M, Oveisi AR, Barkhordar A, Rakhshanipour M, Sargazi-Avval H (2021). Ternary NiCuZr layered double hydroxide@MIL-101 (Fe)-NH_2_ metal-organic framework for photocatalytic degradation of methylene blue. J Nanostruct Chem.

[8] Yang Y, Zhu Q, Peng X, Sun J, Li C, Zhang X (2022). Hydrogels for the removal of the methylene blue dye from wastewater: a review. Environ Chem Lett.

[9] Khasri A, Ahmad MA. Microwave-assisted rubberwood sawdust based activated carbon for adsorption of methylene blue dye: equilibrium, kinetic and thermodynamic studies. AIP Conf Proc. 2019;2124:020022-1-6.

[10] Xue H, Wang X, Xu Q, Dhaouadi F, Sellaoui L, Seliem MK (2022). Adsorption of methylene blue from aqueous solution on activated carbons and composite prepared from an agricultural waste biomass: a comparative study by experimental and advanced modeling analysis. Chem Eng J.

[11] Alim SA, Rao TS, Miditana SR, Lakshmi KVD (2020). Efficient and recyclable visible light-active nickel–phosphorus co-doped TiO_2_ nanocatalysts for the abatement of methylene blue dye. J Nanostruct Chem.

[12] Dutta S, Gupta B, Srivastava SK, Gupta AK (2021). Recent advances on the removal of dyes from wastewater using various adsorbents: a critical review. Mater Adv.

[13] Mashabi RA, Khan ZA, Elwakeel KZ (2022). Chitosan- or glycidyl methacrylate-based adsorbents for the removal of dyes from aqueous solutions: a review. Mater Adv..

[14] Khalili MS, Zare K, Moradi O, Sillanpää M (2018). Preparation and characterization of MWCNT–COOH–cellulose–MgONP nanocomposite as adsorbent for removal of methylene blue from aqueous solutions: isotherm, thermodynamic and kinetic studies. J Nanostruct Chem.

[15] Panda SK, Aggarwal I, Kumar H, Prasad L, Kumar A, Sharma A (2021). Magnetite nanoparticles as sorbents for dye removal: a review. Environ Chem Lett.

[16] Luo JY, Lin YR, Liang BW, Li YD, Mo XW, Zeng QG (2015). Controllable dye adsorption behavior on amorphous tungsten oxide nanosheet surfaces. RSC Adv.

[17] Pérez-González M, Morales-Luna M, Santoyo-Salazar J, Crotte-Ledesma H, García-Tinoco P, Tomás S (2021). Improved adsorption and photocatalytic removal of methylene blue by MoO_3_ thin films: role of the sputtering power, film thickness, and sputtering working pressure. Catal Today.

[18] Zhou W, Deng J, Qin Z, Huang R, Wang Y, Tong S (2022). Construction of MoS_2_ nanoarrays and MoO_3_ nanobelts: two efficient adsorbents for removal of Pb(II), Au(III) and methylene blue. Res J Environ Sci.

[19] Wu Y, Cheng X, Zhang X, Xu Y, Gao S, Zhao H (2017). High efficient and selective removal of Pb^2+^ through formation of lead molybdate on alpha-MoO_3_ porous nanosheets array. J Colloid Interface Sci.

[20] Zhang D, Li J, Liang J, Li H, Yan Y (2018). Self-assembly of α-MoO_3_ flower as a highly effective organics adsorbent for water purification. J Am Ceram Soc.

[21] Ryu S-M, Nam C (2018). Adsorption characteristics of methylene blue on WO_3_ nanorods prepared by microwave-assisted hydrothermal methods. Phys Status Solidi A.

[22] Tong H, Ouyang S, Bi Y, Umezawa N, Oshikiri M, Ye J (2012). Nano-photocatalytic materials: possibilities and challenges. Adv Mater.

[23] Liu X, Jin A, Jia Y, Xia T, Deng C, Zhu M (2017). Synergy of adsorption and visible-light photocatalytic degradation of methylene blue by a bifunctional Z-scheme heterojunction of WO_3_/g-C_3_N_4_. Appl Surf Sci.

[24] Adhikari S, Mandal S, Sarkar D, Kim D-H, Madras G (2017). Kinetics and mechanism of dye adsorption on WO_3_ nanoparticles. Appl Surf Sci.

[25] Galstyan V, Poli N, D'Arco A, Macis S, Lupicd S, Comini E (2020). A novel approach for green synthesis of WO_3_ nanomaterials and their highly selective chemical sensing properties. J Mater Chem A..

[26] Kruse C, Anderson T, Wilson C, Zuhlke C, Alexander D, Gogos G (2013). Extraordinary shifts of the Leidenfrost temperature from multiscale micro/nanostructured surfaces. Langmuir.

[27] Elbahri M, Paretkar D, Hirmas K, Jebril S, Adelung R (2007). Anti-lotus effect for nanostructuring at the leidenfrost temperature. Adv Mater.

[28] Abdelaziz R, Disci-Zayed D, Hedayati MK, Pohls JH, Zillohu AU, Erkartal B (2013). Green chemistry and nanofabrication in a levitated Leidenfrost drop. Nat Commun.

[29] Graeber G, Regulagadda K, Hodel P, Kuttel C, Landolf D, Schutzius TM (2021). Leidenfrost droplet trampolining. Nat Commun.

[30] Leberman R, Soper A (1995). Effect of high salt concentrations on water structure. Nature.

[31] Volta A (1782). XVI. Del modo di render sensibilissima la più debole elettricità sia natural sia artificiale. Philos Trans R Soc.

[32] Lenard P (1915). Über Wasserfallelektrizität und über die Oberflächenbeschaffenheit der Flüssigkeiten. Ann Phys.

[33] Bertus LM, Carcel RA (2011). Prediction of TiO_2_ and WO_3_ nanopowders surface charge by the evaluation of point of zero charge (PZC). Environ Eng Manag J.

[34] Thwala MM, Dlamini LN (2020). Photocatalytic reduction of Cr(VI) using Mg-doped WO_3_ nanoparticles. Environ Technol.

[35] Kgoetlana CM, Malinga SP, Dlamini LN (2020). Photocatalytic degradation of chlorpyrifos with Mn-WO_3_/SnS_2_ heterostructure. Catalysts.

[36] Anik M, Cansizoglu T (2006). Dissolution kinetics of WO_3_ in acidic solutions. J Appl Electrochem.

[37] Liu L, Zhang B, Zhang Y, He Y, Huang L, Tan S (2015). Simultaneous removal of cationic and anionic dyes from environmental water using montmorillonite-pillared graphene oxide. J Chem Eng Data.

[38] Rakass S, Oudghiri Hassani H, Abboudi M, Kooli F, Mohmoud A, Aljuhani A (2018). Molybdenum trioxide: efficient nanosorbent for removal of methylene blue dye from aqueous solutions. Molecules.

[39] Jiang L, Wen Y, Zhu Z, Liu X, Shao W (2021). A Double cross-linked strategy to construct graphene aerogels with highly efficient methylene blue adsorption performance. Chemosphere.

[40] Li H, Budarin VL, Clark JH, North M, Wu X (2022). Rapid and efficient adsorption of methylene blue dye from aqueous solution by hierarchically porous, activated Starbons^®^: mechanism and porosity dependence. J Hazard Mater.

[41] Minamoto C, Fujiwara N, Shigekawa Y, Tada K, Yano J, Yokoyama T (2021). Effect of acidic conditions on decomposition of methylene blue in aqueous solution by air microbubbles. Chemosphere.

[42] Hu D, Li R, Li M, Pei J, Guo F, Zhang S (2018). Photocatalytic efficiencies of WO_3_/TiO_2_ nanoparticles for exhaust decomposition under UV and visible light irradiation. Mater Res Express.

[43] Mahdavi S, Hassani A, Merrikhpour H (2020). Aqueous phosphorous adsorption onto SnO_2_ and WO_3_ nanoparticles in batch mode: kinetic, isotherm and thermodynamic study. J Exp Nanosci.

[44] Dighore N, Dahare P, Gaikwad S, Rajbhoj A (2021). Novel Poly(pyrrole-co-3-acetyl pyrrole)-WO_3_ nanocomposites modified gold electrode as electrocatalytic oxidation and reduction of H_2_O_2_. Adv Mater Lett.

[45] Liu F, Chen X, Xia Q, Tian L, Chen X (2015). Ultrathin tungsten oxide nanowires: oleylamine assisted nonhydrolytic growth, oxygen vacancies and good photocatalytic properties. RSC Adv.

[46] Chithambararaj A, Bose AC (2011). Investigation on structural, thermal, optical and sensing properties of meta-stable hexagonal MoO(3) nanocrystals of one dimensional structure. Beilstein J Nanotechnol.

[47] Gowtham B, Ponnuswamy V, Pradeesh G, Chandrasekaran J, Aradhana D (2018). MoO_3_ overview: hexagonal plate-like MoO_3_ nanoparticles prepared by precipitation method. J Mater Sci Mater Electron.

[48] Pradhan AC, Paul A, Rao GR (2017). Sol–gel-cum-hydrothermal synthesis of mesoporous Co-Fe@Al_2_O_3_−MCM-41 for methylene blue remediation. J Chem Sci.

[49] Osmari TA, Gallon R, Schwaab M, Barbosa-Coutinho E, Severo JB, Pinto JC (2013). Statistical analysis of linear and non-linear regression for the estimation of adsorption isotherm parameters. Adsorp Sci Technol..

[50] Singh K, Senapati K, Sarma K (2017). Synthesis of superparamagnetic Fe_3_O_4_ nanoparticles coated with green tea polyphenols and their use for removal of dye pollutant from aqueous solution. J Environ Chem Eng.

[51] Lagergren S (1898). About the theory of so-called adsorption of solution substances. Handlinge.

[52] Ho YS (2006). Review of second-order models for adsorption systems. J Hazard Mater.

[53] Ys H, Mckay G, Ys H, Mckay GJPB (1999). Pseudo-second order model for sorption processes. Process Biochem.

[54] Noori M, Tahmasebpoor M, Foroutan R (2022). Enhanced adsorption capacity of low-cost magnetic clinoptilolite powders/beads for the effective removal of methylene blue: adsorption and desorption studies. Mater Chem Phys.

[55] El-Bery HM, Saleh M, El-Gendy RA, Saleh MR, Thabet SM (2022). High adsorption capacity of phenol and methylene blue using activated carbon derived from lignocellulosic agriculture wastes. Sci Rep.

[56] Mekuria D, Diro A, Melak F, Asere TG, Rehman R (2022). Adsorptive removal of methylene blue dye using biowaste materials: barley bran and enset midrib leaf. J Chem.

[57] Kulal P, Badalamoole V (2020). Efficient removal of dyes and heavy metal ions from waste water using Gum ghatti–graft–poly(4-acryloylmorpholine) hydrogel incorporated with magnetite nanoparticles. J Environ Chem Eng.

[58] Seera SDK, Kundu D, Gami P, Naik PK, Banerjee T (2021). Synthesis and characterization of xylan-gelatin cross-linked reusable hydrogel for the adsorption of methylene blue. Carbohydr Polym.

[59] Mijinyawa AH, Durga G, Mishra A (2019). A sustainable process for adsorptive removal of methylene blue onto a food grade mucilage: kinetics, thermodynamics, and equilibrium evaluation. Int J Phytoremediat.

[60] Zhang J, Li B, Yang W, Liu J (2014). Synthesis of magnetic Fe_3_O_4_@ hierarchical hollow silica nanospheres for efficient removal of methylene blue from aqueous solutions. Ind Eng Chem Res.

[61] Anushree C, Philip J (2019). Efficient removal of methylene blue dye using cellulose capped Fe_3_O_4_ nanofluids prepared using oxidation-precipitation method. Colloids Surf A Physicochem Eng Asp.

[62] Tarekegn MM, Balakrishnan RM, Hiruy AM, Dekebo AH (2021). Removal of methylene blue dye using nano zerovalent iron, nanoclay and iron impregnated nanoclay—a comparative study. RSC Adv.

